# Ameliorating Uniformity and Color Conversion Efficiency in Quantum Dot-Based Micro-LED Displays through Blue–UV Hybrid Structures

**DOI:** 10.3390/nano13142099

**Published:** 2023-07-19

**Authors:** Tzu-Yi Lee, Wen-Chien Miao, Yu-Ying Hung, Yi-Hong Bai, Pei-Tien Chen, Wei-Ta Huang, Kuan-An Chen, Chien-Chung Lin, Fang-Chung Chen, Yu-Heng Hong, Hao-Chung Kuo

**Affiliations:** 1 Department of Photonics, College of Electrical and Computer Engineering, National Yang Ming Chiao Tung University, Hsinchu 30010, Taiwan; alex860512.ee10@nycu.edu.tw (T.-Y.L.); white.white.ee11@nycu.edu.tw (Y.-Y.H.); johnny0970381255.ee10@nycu.edu.tw (Y.-H.B.); dy1998416.ee12@nycu.edu.tw (P.-T.C.); aaronhuang.c@nycu.edu.tw (W.-T.H.); fcchendop@nycu.edu.tw (F.-C.C.); 2 Semiconductor Research Center, Hon Hai Research Institute, Taipei 11492, Taiwan; leona.wc.miao@foxconn.com; 3 Department of Electrophysics, College of Science, National Yang Ming Chiao Tung University, Hsinchu 30010, Taiwan; 4 SynthEdge Advanced Materials Corp. Ltd., Taoyuan 32742, Taiwan; mageo.chen@synth-edge.com; 5 Graduate Institute of Photonics and Optoelectronics, National Taiwan University, Taipei 10617, Taiwan; chienchunglin@ntu.edu.tw

**Keywords:** quantum dot color conversion layer, ALD passivation technology, micro-LED, intermixing quantum well, modified DBR

## Abstract

Quantum dot (QD)-based RGB micro light-emitting diode (μ-LED) technology shows immense potential for achieving full-color displays. In this study, we propose a novel structural design that combines blue and quantum well (QW)-intermixing ultraviolet (UV)-hybrid μ-LEDs to achieve high color-conversion efficiency (CCE). For the first time, the impact of various combinations of QD and TiO_2_ concentrations, as well as thickness variations on photoluminescence efficiency (PLQY), has been systematically examined through simulation. High-efficiency color-conversion layer (CCL) have been successfully fabricated as a result of these simulations, leading to significant savings in time and material costs. By incorporating scattering particles of TiO_2_ in the CCL, we successfully scatter light and disperse QDs, effectively reducing self-aggregation and greatly improving illumination uniformity. Additionally, this design significantly enhances light absorption within the QD films. To enhance device reliability, we introduce a passivation protection layer using low-temperature atomic layer deposition (ALD) technology on the CCL surface. Moreover, we achieve impressive CCE values of 96.25% and 92.91% for the red and green CCLs, respectively, by integrating a modified distributed Bragg reflector (DBR) to suppress light leakage. Our hybrid structure design, in combination with an optical simulation system, not only facilitates rapid acquisition of optimal parameters for highly uniform and efficient color conversion in μ-LED displays but also expands the color gamut to achieve 128.2% in the National Television Standards Committee (NTSC) space and 95.8% in the Rec. 2020 standard. In essence, this research outlines a promising avenue towards the development of bespoke, high-performance μ-LED displays.

## 1. Introduction

The rise of the metaverse has sparked a rapid evolution in display technology, driving the need for smaller, more versatile devices to cater to the demands of wearable applications [1,2,3]. Thereby, micro light-emitting diodes (µ-LEDs) have emerged as a promising display technology, comprising millions of LEDs with dimensions ranging from tens to hundreds of micrometers. Compared to traditional liquid crystal displays (LCDs) and organic light-emitting diodes (OLEDs), µ-LEDs offer a host of advantages, including high brightness, high refresh rates, wide viewing angles, low power consumption, and a wide color gamut, positioning them as the future choice for display technology [3,4,5]. However, despite their potential, µ-LED technology still faces significant challenges [6,7,8]. Specifically, red µ-LED wafers encounter hurdles related to performance and transfer yields, impeding large-scale production and commercialization [3,9,10]. Overcoming these obstacles is crucial for the widespread adoption of µ-LEDs displays in various applications, including the metaverse. In this ever-evolving landscape, researchers and industry experts are diligently working to address these issues and unlock the full potential of µ-LED technology. By surmounting these challenges, µ-LED displays will pave the way for immersive, high-quality visual experiences in the metaverse and beyond.

Quantum dot (QD) phosphors demonstrate strong absorption of ultraviolet (UV) and blue light, coupled with high color-conversion efficiency (CCE), making them suitable for use as color-conversion layers (CCLs). Additionally, they have a narrow full width at half maximum (FWHM), which can effectively enhance color purity and result in a broader color gamut. Hence, the integration of blue µ-LED s with QD color conversion technology is viewed as one of the effective alternatives today for overcoming challenges associated with RGB tri-color LED transfer technology [10,11,12,13,14,15]. In 2020, Kuo et al. presented a full-color µ-LED fabricated from a semi-polar chip with a QD photoresist CCL [16]. The semi-polar chip not only exhibited improved wavelength stability but also achieved a wide color gamut of 114.4% national television standards committee (NTSC) through QD implementation. Yin et al. utilized a spray coating technique to deposit approximately 10 µm of CCL on µ-LEDs and produced a full-color display [17]. However, the Achilles’ heel of QDs is their reliability due to their lower formation energy, which makes them vulnerable to external factors, like water and oxygen [18,19,20]. In the absence of a protective layer, QDs undergo rapid quenching, leading to a substantial decline in photoluminescence quantum yield (PLQY). Therefore, incorporating a passivation protection layer is crucial for CCLs [21]. Lee et al. employed atomic layer deposition (ALD) passivation techniques to coat the QD surface with Al_2_O_3_, demonstrating impressive reliability in long-term aging and high temperature and humidity tests [22]. Similarly, Kuo et al. leveraged low-temperature ALD technology to deposit Alumina (Al_2_O_3_) on the QD film surface of the CCL, effectively obstructing water and oxygen and enhancing reliability [9,23]. Lin et al. also used ALD passivation protection to allow QD CCLs to be stored at normal room temperature for more than 9000 h with a linear extrapolation of 44,041 h [10].

**Figure 1:**
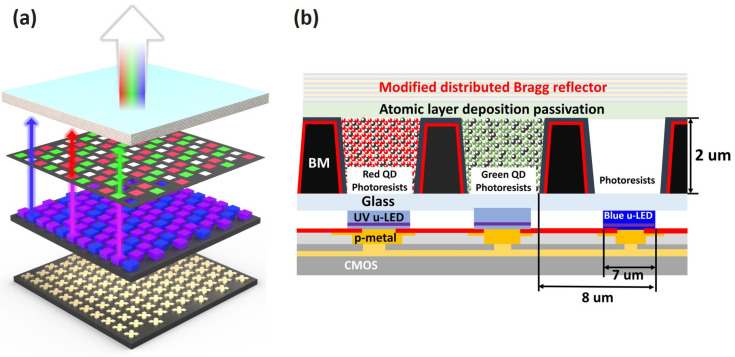
(**a**) Schematic diagram of a full-color display with blue–UV light mixing. (**b**) Cross-sectional view of a single RGB pixel.

As display device sizes continue to diminish and become thinner, the QD CCL often suffers from insufficient thickness, resulting in substantial leakage of blue light. This leads to a significant decrease in CCE. One solution to this problem is to embed QDs into nano-porous µ-LED s, a structure that allows for QD accommodation and enhances light scattering, thus mitigating blue-light leakage [23]. Adding nanoscale scattering particles to elongate the light path, thereby boosting CCE and light intensity, is also a compelling strategy [14,17,24]. Common scattering particles include BN, SiO_2_, Al_2_O_3_, ZrO_2_, TiO_2_, and organic particles, among others. TiO_2_, with the highest refractive index (2.76~2.55), is the most widely used scattering material. To achieve the best scattering and reflection effects in the color conversion layer, it is not only important to select TiO_2_ particles with a high refractive index and pair them with plastic materials that have a significantly different refractive index for efficient reflection and refraction effects, but also to consider the impact of diffraction. If the content of TiO_2_ particles is increased to improve reflectivity, the particles can become overly crowded, leading to a poor diffraction effect. Besides the light deterioration caused by increased content, the agglomeration of TiO_2_ particles can also negatively affect light performance. Therefore, ensuring the effective dispersion of TiO_2_ particles is a crucial factor for the performance of the color conversion layer. Furthermore, employing a distributed Bragg reflector (DBR) to reflect the excitation light source has become a prevalent method to enhance CCE [9,10,11,22,25]. Given that QDs exhibit a higher absorption of UV light, an alternate strategy could involve substituting blue µ-LED s with UV light to further enhance CCE. Coupling this with a DBR to reflect leaked UV light back into the CCL can result in a significant increase in luminous flux [11,12]. However, the PLQY and stability of blue QDs are currently suboptimal, leaving much room for improvement in practical applications. As a response to this, our study presents a hybrid µ-LED array based on quantum well (QW) intermixing UV [26] and blue light. The array’s individual pixel size is 7 × 7 µm^2^ with a pitch of 1 µm. This design incorporates red and green QDs and TiO_2_ QD photoresists (QDPRs) on QW intermixing UV µ-LEDs, using a modified DBR to reclaim excess UV light. The incorporation of TiO_2_ not only elevates the CCE but also addresses issues of uniformity. Consequently, we realize a full-color µ-LED array, offering a viable approach for the development of highly uniform color conversion µ-LED displays. The complete structure is shown schematically in Figure 1.

## 2. Simulated Method and Structure Definition

A comprehensive numerical study was conducted using LightTools (8.6.0) lighting design software. Initially, a rectangular structure with a thickness of 0.3 μm was modeled. The primary structure includes the substrate and the receiving plane. The external excitation sources are UV and blue μ-LEDs. These LEDs are modeled with a Gaussian spectral distribution over a surface area of 7 × 7 μm^2^, peak wavelengths of 410 nm and 450 nm, respectively, and a FWHM setting of 20 nm. In this model, there are four primary types of optical events when light is emitted from the LED to the receiver plane:

(1) Transmission through the DBR structure to the receiver plane: Light from the LEDs strikes the DBR structure at an angle that does not achieve total internal reflection; Therefore, it follows Snell’s law and reaches the receiver plane through refraction.
(2) Direct to Receiver Plane: The light generated by the LED is directed straight to the receiver plane, bypassing any objects;
(3) Reflected light from DBR structure: Reflected light is produced when the angle of incidence on the DBR reaches the total internal reflection (TIR) angle, and it subsequently reaches the receiver plane;
(4) Reflected light from the receiver plane: Reflected light is produced when light impinges upon the receiver plane.

Due to the aforementioned four types of optical events, multiple light reflections occur between the receiver plane, the DBR, and the substrate. Unless these reflections strike a perfectly reflective surface, energy loss occurs within the optical system. To minimize this loss, the substrate surface is coated with a high reflectivity coating in the module design.

The CCL module is set up as follows: The QDPR consists of either red or green QDs mixed with TiO_2_ scattering particles. We have assumed a uniform distribution of all particles within the film. The particle sizes for red and green QDs in the QDPR were fixed at 25 nm and 20 nm with emission wavelengths of 625 nm and 535 nm, respectively. Their respective FWHMs were set at 25 nm and 24 nm, respectively. Additionally, the absorption spectra of both the red and green QDs were imported into the QDPR. The particle size of TiO_2_ was established as 250 nm. Finally, the refractive index of the PR was set at 1.47. A 150 μm high transparent glass layer (with a light transmittance of 91.6%) [27] was placed between the QDPR and the UV or blue-light μ-LED. The QDPR shares the same light-receiving area as the excitation source, ensuring that light emitted from the UV or blue μ-LED is completely converted into green or red light upon passing through the red and green QDPRs. The array structure was modeled as a 6 × 6 μ-LED array with a black matrix (BM) serving as a blocking wall. This barrier wall, made primarily of high-absorption material, was introduced to reduce optical crosstalk. Lastly, a modified DBR was created atop the QDPR, with almost 100% reflectivity for UV light, significantly reducing UV light leakage from the structure to improve the CCE. In this simulation, the impacts of varying QD concentrations (10–70%), TiO_2_ concentrations (0–35%), and QDPR thicknesses (1–10 μm) on PLQY, CCE, and homogeneity were explored. The difference in light leakage was compared before and after the addition of the modified DBR.

## 3. Experiment and Fabrication Process

### 3.1. UV and Blue μ-LED Preparation Process

In this study, we successfully developed a high-performance, full-color-emitting QD μ-LED array. We first fabricated UV and blue μ-LED s with a size of 7 × 7 µm^2^, arranged into a 6 × 6 array. Epitaxial structures for UV and blue μ-LEDs are grown on a patterned sapphire substrate (PSS) oriented at (22–43) via metal organic vapor deposition (MOCVD). This substrate features an undoped GaN buffer layer and n-type GaN. The active region is composed of n-GaN layers, InGaN/GaN multiple quantum wells (MQWs), and a p-type GaN layer. The resulting emission wavelength is set at 450 nm. Selected area annealing was performed on the constituents. The UV μ-LED samples were coated with a molybdenum/SiO_2_ capping on the surface, while the blue μ-LEDs were coated with SiO_2_ capping. These samples were then annealed in N_2_ at 950 °C in a filled repetitive rapid thermal annealing (RRTA) chamber, undergoing two 120 s cycles. This was followed by the application of p-type GaN and the current diffusion layer for metal contact. A tin oxide (ITO) layer is deposited atop the p-GaN layer. Electrodes composed of Ti/Al/Ti/Au, deposited to a thickness of 20/125/45/75 nm, respectively, are used. The device’s passivation layer is achieved through the atomic layer deposition (ALD) of Al_2_O_3_/SiO_2_. Finally, Ti/Al/Au with a deposition thickness of 20/250/300 nm, respectively, is employed as the metal solder pad. The μ-LED array was fabricated as follows: First, we etched the n-type GaN using the inductively coupled plasma etching technique. Then, we deposited p-type metal on the current diffusion layer and n-type metal on the n-type GaN layer. This was followed by inductively coupled plasma etching to carve grooves in the sapphire substrate, thus ensuring the isolation of the μ-LEDs. Finally, we completed the fabrication of the μ-LED array by depositing passivated SiO_2_ on the sidewalls through ALD technology.

### 3.2. QDPR Array Preparation Technology

**Figure 2:**
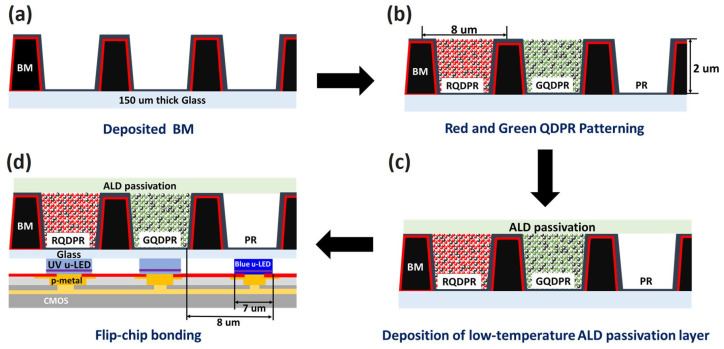
Schematic diagrams of the photoresist process for creating a black PR matrix and QDPR on a hybrid μ-LED array. (**a**) Deposition of BM on a glass substrate, (**b**) Sequential deposition of red and green QDPR, (**c**) Application of an Al_2_O_3_ protective layer on the CCL using a low-temperature ALD technique, and (**d**) Bonding of the CCL with the μ-LED array.

Figure 2 provides a schematic representation of the photoresist process employed to create a black PR matrix and QDPR on a hybrid μ-LED array. Initially, a black PR layer, 2 μm thick, is deposited on a highly transparent glass substrate, which has a thickness of 150 μm. The black matrix (BM) serves two purposes: it flattens the μ-LED array and prevents horizontal blue light leakage. This is depicted in Figure 2a. Subsequently, red QDPR, green QDPR, and transparent PR are prepared sequentially using the photolithography process to form color pixels, as illustrated in Figure 2b. Each color pixel is designed with dimensions of 7 × 7 μm^2^ and a 1 μm space is left between each pixel. Then, the QD is passivated by depositing Al_2_O_3_ onto the QDPR array through a low-temperature ALD technique (50 °C). This step, shown in Figure 2c, shields the QDs from high temperatures and potential alterations in material properties while also protecting them against humidity and oxidation. Ultimately, as shown in Figure 2d, the colored pixel arrays on the glass are bonded to the μ-LED arrays using aligners and UV resins.

## 4. Result and Discussion

**Figure 3:**
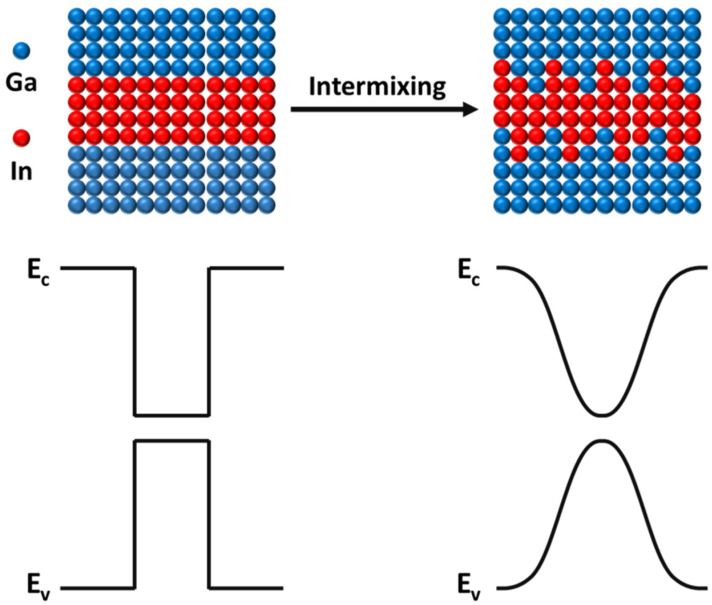
Schematic diagram of the energy band of QW mixing when the input current density is equal to 1500 A/cm^2^.

For display applications, the main issues with LEDs are unstable wavelengths, wide FWHM, and broad divergence angles. Unstable wavelengths and wide FWHMs can result in color variations across the display, diminishing the image’s vibrancy and sharpness. The quantum confinement Stark effect (QCSE), primarily caused by lattice mismatch in InGaN/GaN QWs, is one such problem [21,28,29]. The lattice mismatch triggers a piezoelectric field that skews the energy band edges, causing electrons and holes to spatially separate and thereby reducing the electron–hole wave function overlap [30,31]. As the indium content in the InGaN QW increases, the QCSE becomes more pronounced, which further restricts the efficiency of µ-LEDs. Post-growth annealing is an effective method to create an InGaN/GaN QW with a softened potential distribution [26,32]. This process facilitates the interdiffusion of group III atomic species from the QW to adjacent quantum barriers (QBs), forming gradient composition interfaces, as depicted in Figure 3. We employ a high spatial resolution and region-selective hybrid approach to accomplish the potential engineering of InGaN/GaN QW LEDs while preserving high material quality. This is achieved through a combination of a metal/dielectric overlay and RRTA at temperatures below 1000 °C.

**Figure 4:**
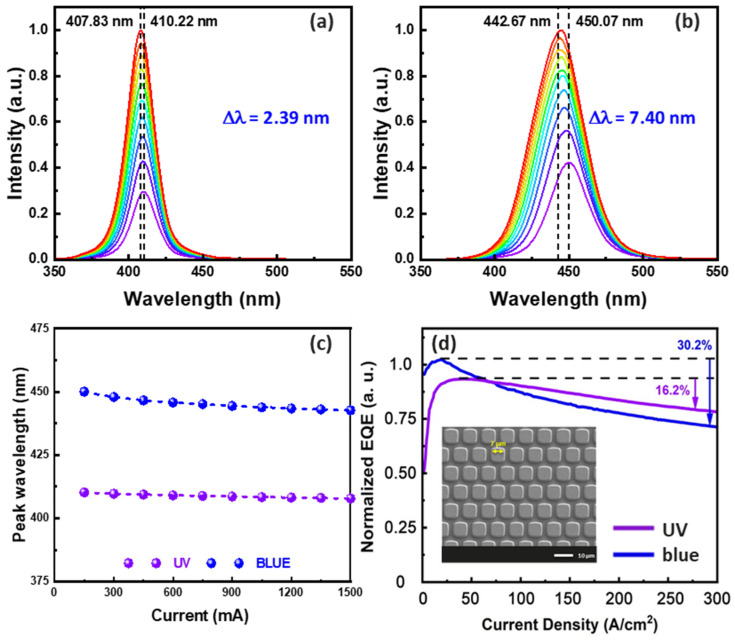
(**a**) UV EL spectrum of the QW intermixing. (**b**) Blue EL spectrum of conventional MQW. Comparison of conventional MQW blue light with QW intermixing UV μ-LED. (**c**) Wavelength shift; (**d**) EQE (the inset shows a scanning electron microscope (SEM) image of a blue-UV hybrid μ-LED, where the scale is 10 μm).

Optical and electrical properties of μ-LED devices under direct current were measured using a spectrometer (Maya 2000 Pro), where the emitted light was collected by an integrating sphere. When the input current density of the UV μ-LED using QW intermixing increased from 150 A/cm^2^ to 1500 A/cm^2^, the peak wavelength changed from 410.22 nm to 407.83 nm, corresponding to a blue shift of 2.39 nm. In contrast, the peak wavelength of the conventional multiple quantum well (MQW) blue μ-LED changed from 450.07 nm to 442.67 nm, exhibiting a blue shift of 7.04 nm. Consequently, the wavelength shift of the UV μ-LED is smaller under varying current measurements, as shown in Figure 4a–c. Regarding external quantum efficiency (EQE) performance, UV μ-LEDs display superior sag characteristics, as depicted in Figure 4d. This is due to the QCSE in the polar c-plane GaN, which results in an efficiency drop at low current densities. Nonetheless, thanks to the presence of a three-layer interleaved QW structure, the device experiences less efficiency degradation compared to conventional MQW blues, improving the conversion efficiency at higher injection currents [26].

**Figure 5:**
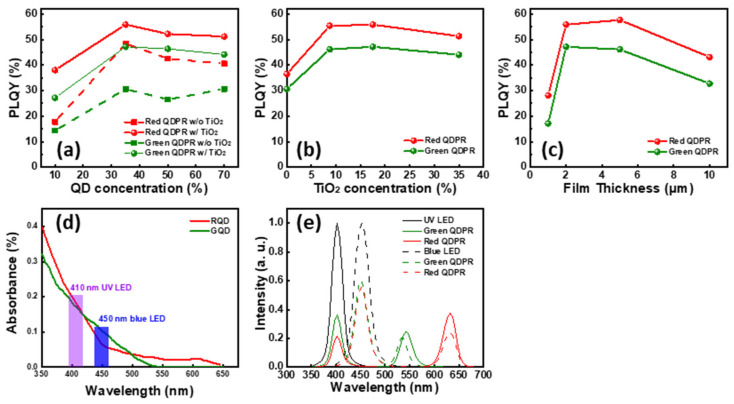
Simulation of the effects of (**a**) QD concentration, (**b**) TiO_2_ concentration, and (**c**) film thickness on the red and green QDPR PLQY. (**d**) Red and green QD absorption spectra. (**e**) Comparison of the effect of UV and blue light on PLQY.

To realize the application of a full-color microdisplay. We perform optical simulations for the characteristics of QD. In this study, we first investigated the effects of different QD concentrations (10–70%), TiO_2_ concentrations (0–35%), and QDPR thicknesses (1–10 μm) on the PLQY using LightTools (8.6.0) lighting design software, as depicted in Figure 5a–c. We observed the highest PLQY when the QD concentration in the QDPR was increased from 10% to 35%. Beyond this point, increasing the concentration resulted in a gradual decline in PLQY. This decline can be attributed to the self-agglomeration phenomenon that occurs when the QD concentration is excessively high, which adversely impacts the PLQY [14,23,24]. Interestingly, the judicious addition of scattering particles such as TiO_2_ can help alleviate this issue. TiO_2_ not only increases the optical path of QD, thereby enabling lights to remain in the film for a longer duration, but it also increases the separation between QDs. This enhancement helps mitigate the self-agglomeration of QDs, thereby effectively increasing the PLQY. Typically, TiO_2_ particles serving as scattering centers should have a diameter in the submicron range. If the scattering particles are too small, they will not scatter the incident light effectively. Given that our CCLs are only a few micrometers thick, scattering particles that are too large could lead to uneven dispersion on the film’s surface. Thus, selecting an appropriate particle size is of utmost importance. In this study, we chose to maintain a consistent TiO_2_ particle size of approximately 250 nm. Moreover, we found that the thickness of the QDPR also impacts the PLQY. We had to select an appropriate QD and TiO_2_ concentration to optimize the thickness. If the QDPR is too thin, significant light leakage can occur, leading to a low PLQY [33]. As demonstrated in Figure 5d, compared to blue light, QDs absorb UV light more effectively. Consequently, using UV light as an excitation source can lead to higher color conversion efficiency. Figure 5e further substantiates this point, illustrating that under the same film formation conditions for the QDPR, UV light has a superior color conversion capability.

**Figure 6:**
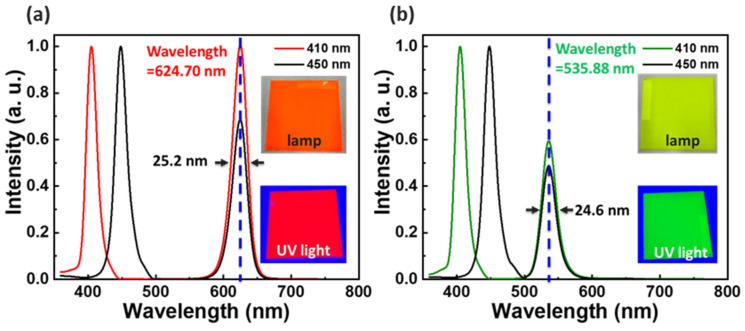
Comparison of actual CCL with UV and blue light in the EL. (**a**) Red. (**b**) Green.

Based on the simulation results, a QDPR paste ratio of 35% QD concentration and 17.5% TiO_2_, and a film thickness of 2 μm was selected. This QDPR paste was applied to a highly translucent glass substrate by rotating it at 220 rpm. Subsequently, the QDPR CCL was produced using a photolithography process. We used a fixed 1 W light source for excitation, with UV and blue light as the excitation wavelengths. The PLQY was calculated using electroluminescence (EL) spectroscopy, as depicted in Figure 6. Under UV excitation, the PLQY for the red and green QDPR was 57.8% and 45.0%, respectively. When excited with blue light, the PLQY values were 44.4% and 36.3%, respectively.

**Figure 7:**
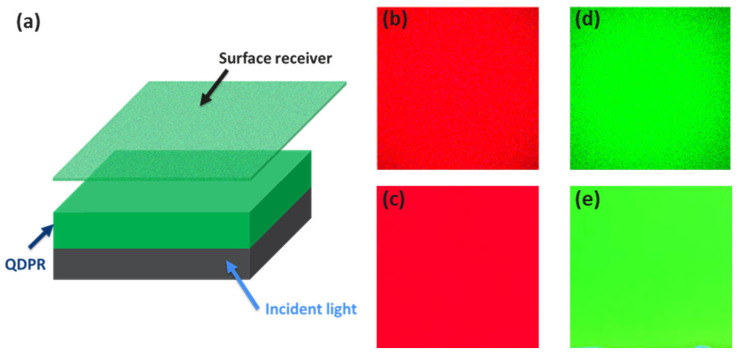
(**a**) A LightTools model featuring a surface receiver, QDPR structure, and light source. with simulated luminescence images in QD for (**b**) red and (**d**) green. The measured FLOM for (**c**) red and (**e**) green.

Due to the self-aggregation effect of QDs, the illumination uniformity of the QD photolithography process is subpar. However, in our proposed structure, the addition of scattering particles amplifies the light scattering effect, thereby increasing the possibility of blue-light-stimulating QDs. Moreover, incorporating TiO_2_ can maintain the position of the QDs to mitigate the self-aggregation effect after the QD rotational coating process, which in turn improves color performance. Figure 7b–d simulate the uniformity of red and green luminescence in structures with TiO_2_. The illumination uniformity of the red and green QDPRs is 95.2% and 91.1%, respectively. Figure 7c and e depict the red and green luminescence images for structures with TiO_2_. To analyze the color performance of the luminous images, fluorescent luminescent optical microscope (FLOM) images were converted to grayscale and divided into 300 × 300 pixels using MATLAB. Illuminance uniformity is defined as the ratio between the minimum value of illuminance pixels and the average value of illuminance pixels [23]. The illuminance uniformity of the red and green QDPRs is 98.6% and 96.7%, respectively. The luminous images and illuminance uniformity are in good agreement with the measured data. Consequently, the QD self-aggregation phenomenon is effectively suppressed, yielding high color purity, which holds great promise for display technology.

**Figure 8:**
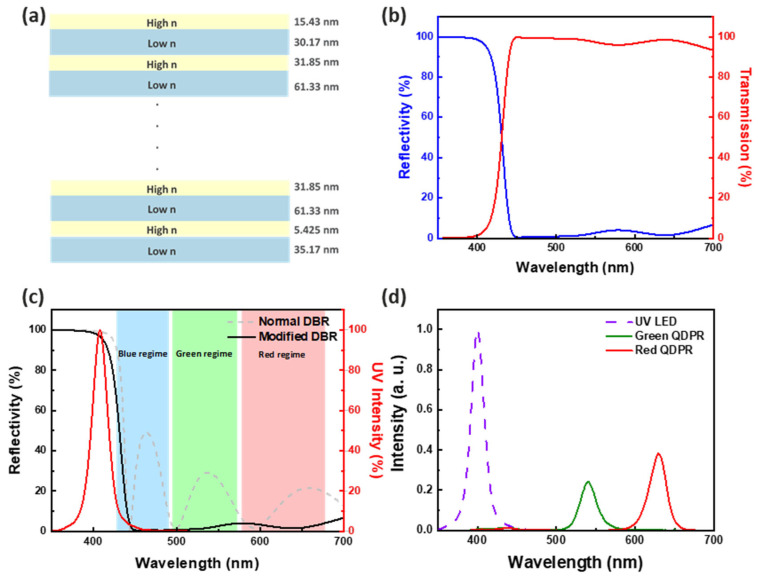
(**a**) Schematic diagram of the modified DBR structure. (**b**) Calculation of transmittance and reflectance spectra of modified DBR structures. (**c**) Comparison of the reflectance calculated spectra of normal DBR structure and modified DBR structure for the emissive wavelength of 410 nm. (**d**) Red and green QDPR plus improved EL spectra of DBR.

Although the use of QW-intermixing UV μ-LED as the excitation source for QD CCL can effectively enhance the device’s PLQY, the light leakage phenomenon cannot be completely avoided when utilizing a thinner film. The CCE can be used to evaluate this leakage, as it is a key photonic characteristic of the CCL. Pump LEDs output high-energy photons (in the blue or UV region) that are then absorbed by the active material inside the CCL and re-emitted as low-energy photons [10,22]. Therefore, to further suppress the light leakage, we designed a modified DBR on top of the overall structure. This is a reflector comprised of multiple alternating layers of materials with different refractive indices. In a multilayer structure with periodic contrast of high and low optical refractive indices, when each layer’s thickness equals one-fourth of the wavelength, partial reflection at each surface combines with phase length interference, allowing the multilayer to act as a high-quality, adjustable reflector in terms of reflectivity and bandwidth [25,34]. Ideally, a flat transmission spectrum with over 90% transmittance in the long wavelength range is required. We designed the modified DBR structure as the first pair of SiO_2_/TiO_2_ (35.165 nm/5.4250 nm) and the last pair of SiO_2_/TiO_2_ (30.165 nm/15.425 nm) for the calculated transmittance and reflectance spectra, as shown in Figure 8a,b. Compared to a conventional DBR, the modified DBR can reduce or even eliminate unwanted ripples, as shown in Figure 8c. Figure 8d demonstrates the EL of the modified DBR over the red and green QDPR, showing effective suppression of the UV. The CCE calculation by EL reveals that the CCE of red and green QDPR is 96.25% and 92.91%, respectively. This indicates that our improved DBR can effectively suppress UV light leakage without losing significant QD radiation.

**Figure 9:**
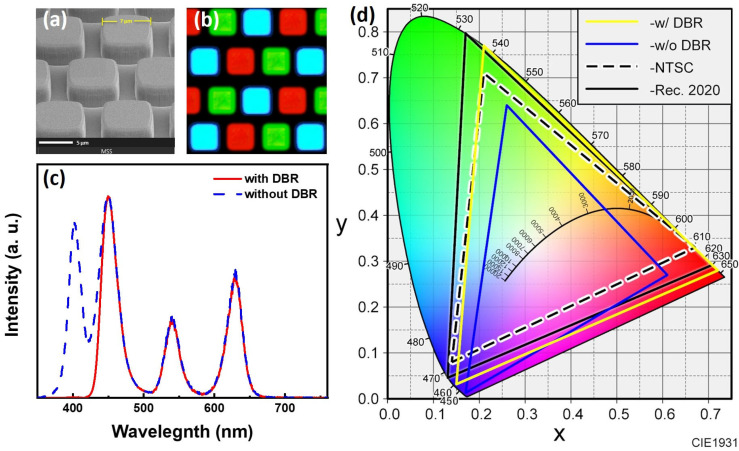
(**a**) SEM image of the blue–UV hybrid μ-LED array. (**b**) ELOM image. (**c**) CIE-1931 chromaticity map of RGB μ-LED pixels. (**d**) The CIE-1931 chromaticity diagram of RGB pixel μ-LED.

We fabricated a blue–UV hybrid μ-LED array with a single pixel size of 7 × 7 μm^2^ and a spacing of 1 μm. Notably, we applied a protective layer of Al_2_O_3_ atop the QDPR array using a low-temperature ALD technique. From previous studies, it has been demonstrated that the oxide coating on the QD surface using the ALD passivation protection technique can effectively protect QDs from high temperatures, changes in material properties, and environmental factors, such as humidity and oxidation [10,22,23,24]. Figure 9a presents an SEM image of a blue–UV light hybrid μ-LED. Figure 9b, however, displays an electroluminescence optical microscope (ELOM) image of the QDPR array with blue–UV hybrid μ-LEDs serving as the excitation source. The results reveal that the QDPR demonstrates excellent uniformity. Figure 9c showcases the spectral changes after the addition of the modified DBR, which was measured via EL and received by an integrated sphere. The optical properties are superb after incorporating the modified DBR, which can effectively suppress the leakage of UV light without sacrificing the RGB tri-color. In the CIE-1931 colorimetric chart, the color gamut area of the unmodified DBR part is 79.9% and 59.6% according to the NTSC and Rec. 2020 colorimetric space standards, respectively. With the addition of the modified DBR, the gamut area increases to 128.2% and 95.8%, as shown in Figure 9d. This significant improvement in light leakage allows for the display of thin QD-based CCLs with high color purity.

## 5. Conclusions

Our study successfully exhibits the superior optical properties of a high photoluminescence QDPR array with incorporated blue and quantum-well-intermixing UV hybrid μ-LEDs. This unique approach overcomes QD self-aggregation while achieving extraordinary illumination uniformity, with both red and green subpixels demonstrating an exceptional uniformity of 98.6% and 96.7%, respectively. The CCL is constructed by depositing colloidal QDs onto a highly translucent glass substrate using photolithography. With excitation from 410 nm UV light, the red and green QDPRs achieve notable PLQY of 57.8% and 45.0%, respectively. To ensure QD stability and longevity, we employ a low-temperature ALD passivation protection technology on the CCL. The integration of a modified DBR suppresses light leakage, enhancing the CCE of the red and green CCLs, reaching values of 96.25% and 92.91%, respectively. The incorporation of the modified DBR broadens the color gamut, achieving expansive color range with 128.2% coverage in the NTSC space and 95.8% in the Rec. 2020 standard. This combination of high-uniformity hybrid μ-LEDs and high-PLQY QDPR arrays opens up exciting opportunities for future display technologies, enhancing color performance and uniformity.

## Data Availability

All data generated or analyzed during this work are included in this published article.
